# Polybrominated Diphenyl Ether Exposure and Thyroid Function Tests in North American Adults

**DOI:** 10.1289/ehp.1509755

**Published:** 2015-09-15

**Authors:** Colleen M. Makey, Michael D. McClean, Lewis E. Braverman, Elizabeth N. Pearce, Xue-Mei He, Andreas Sjödin, Janice M. Weinberg, Thomas F. Webster

**Affiliations:** 1 Department of Environmental Health, Boston University School of Public Health, Boston, Massachusetts, USA; 2 Section of Endocrinology, Diabetes, and Nutrition, Boston University School of Medicine, Boston, Massachusetts, USA; 3 National Center for Environmental Health, Centers for Disease Control and Prevention, Atlanta, Georgia, USA; 4 Department of Biostatistics, Boston University School of Public Health, Boston, Massachusetts, USA

## Abstract

**Background:**

Polybrominated diphenyl ethers (PBDEs) are flame-retardant chemicals that are added to many consumer products. Multiple animal studies have shown PBDEs to be thyroid hormone (TH) disruptors. Epidemiologic evidence of PBDE exposure associated with TH disruption has been inconclusive.

**Objectives:**

We used repeated measures to estimate associations between serum PBDE concentrations and THs in a North American adult cohort.

**Methods:**

From 2010 to 2011, we collected ≤ 3 serum samples at approximately 6-month intervals from 52 healthy adult office workers from Boston, Massachusetts, for analysis of PBDE congeners and THs.

**Results:**

The geometric mean sum concentrations of the most prevalent PBDE congeners (BDE-28, BDE-47, BDE-99, BDE-100, and BDE-153) were 22 ng/g lipid in winter 2010, 23 ng/g lipid in summer 2010, and 19 ng/g lipid in winter 2011. BDE-47 was the predominant congener. Based on a multivariable mixed regression model, we estimated that on average, a 1-ng/g serum increase in BDE-47 was associated with a 2.6-μg/dL decrease in total thyroxine (T4) (95% CI: –4.7, –0.35). Total T4 was inversely associated with each PBDE congener. Serum concentrations of PBDEs were not strongly associated with total triiodothyronine (T3), free T4, or thyroid-stimulating hormone (TSH).

**Conclusion:**

These results are consistent with those from animal studies showing that exposure to PBDEs is associated with a decrease in serum T4. Because the other TH concentrations did not appear to be associated with BDE exposures, our findings do not indicate effects on the pituitary–thyroid axis. Taken together, our findings suggest that PBDE exposure might decrease the binding of T4 to serum T4 binding proteins.

**Citation:**

Makey CM, McClean MD, Braverman LE, Pearce EN, He XM, Sjödin A, Weinberg JM, Webster TF. 2016. Polybrominated diphenyl ether exposure and thyroid function tests in North American adults. Environ Health Perspect 124:420–425; http://dx.doi.org/10.1289/ehp.1509755

## Introduction

Polybrominated diphenyl ethers (PBDEs) have been used since the 1970s as additive flame retardants. The technical formulation pentabromodiphenyl ether (pentaBDE), composed of PBDE congeners containing three to six bromines, was added primarily to products containing polyurethane foam. Owing to their persistence in the environment, ability to bioaccumulate, and potential adverse health effects, pentaBDEs were added to the list of persistent organic pollutants in the Stockholm Convention in 2004 (Stockholm Convention 2004). U.S. manufacturers of pentaBDE and octabromodiphenyl ether (octaBDE) voluntarily halted production in 2004. Nevertheless, PBDEs continue to be found in food products (Schecter et al. 2010) and indoor microenvironments (e.g., homes, offices, vehicles) (Watkins et al. 2012). Therefore, it is likely that people in the United States continue to be exposed to PBDEs through exposures to indoor dust and diet (Fraser et al. 2009; Watkins et al. 2012).

Animal studies have established that pentaBDEs are endocrine-disrupting chemicals, which can modify thyroid hormone (TH) levels (Dishaw et al. 2014; Fowles et al. 1994; Stoker et al. 2004; Zhou et al. 2002). PentaBDEs and their cytochrome P450 (CYP450)–mediated metabolites, hydroxylated pentaBDEs (OH-PBDEs), are structurally similar to thyroxine (T_4_) and triiodothyronine (T_3_) (Meerts et al. 2000). Multiple experimental studies in rodents have shown that exposure to these pentaBDE congeners leads to hypothyroxinemia, characterized by a decrease in serum total T_4_ (TT_4_) concentrations (Fowles et al. 1994; Stoker et al. 2004; Zhou et al. 2002).

In humans, THs are essential for proper growth and development *in utero* and during infancy, as well as for maintenance of many organ systems and metabolism throughout life (Cooper and Biondi 2012). Although the toxicological evidence of PBDEs causing thyroid disruption is robust, the epidemiologic evidence has been inconsistent, with positive, negative, and null associations reported between PBDE exposures and THs (Abdelouahab et al. 2013; Bloom et al. 2008; Chevrier et al. 2011; Stapleton et al. 2011; Turyk et al. 2008; Zota et al. 2011).

In the present study, we used repeated serum measures to assess the association between PBDE exposure and thyroid function in a longitudinal cohort of healthy adults. Our primary aim was to use these data to examine the association between PBDEs and thyroid function tests (TFTs) by measuring total T_4_ (TT_4_,) free thyroxine (fT_4_), total T_3_ (TT_3_), and thyroid-stimulating hormone (TSH). We also evaluated confounding and effect modification of these associations in our cohort.

## Methods

*Study design and population.* We recruited 26 male and 26 female adult office workers living in the Boston (Massachusetts) metropolitan area and collected serum samples at approximately 6-month intervals from January 2010 to May 2011. This study is part of an extensive study of the effects of exposure to flame retardants on the health of office workers. Eligible subjects were required to be nonsmoking adults over the age of 18, self-described as generally healthy, and planning to reside in the Boston Metropolitan area for the duration of the study. Participants were excluded for having a prior diagnosis of thyroid disease or if they were pregnant. Characteristics and descriptions of the Flame Retardant Exposure (FlaRE) study population have been presented elsewhere (Makey et al. 2014). Forty-one participants completed all three study visits, 9 completed two study visits and 2 completed only one study visit (total of 143 serum samples). There were 4 participants (total of 6 serum samples) with serum samples excluded from analysis. The reasons for exclusion were as follows: use of a thyroid-affecting medication (1 participant, 3 serum samples), pregnancy at the third sampling round (1 serum sample), inadequate serum volume at the first sampling round (1 serum sample), and suspected field contamination at the third sampling round (1 serum sample). The contamination of 1 sample in the field was suspected because the concentration of hexabromodiphenyl ethers (hexaBDEs) in that sample was 10 times higher than in the other 2 samples collected from the same participant, whereas lower brominated congeners were comparable among the 3 samples, suggesting sample contamination with residential dust containing the octaBDE technical mixture. Thus, the present study used 137 PBDE and hormone measures in serum collected from 51 participants. We obtained written informed consent before participation, and the Boston University Medical Center Institutional Review Board approved the study protocol. The involvement of the Centers for Disease Control and Prevention (CDC) did not constitute engagement in human subjects research.

*Blood samples.* A trained phlebotomist collected 30 mL of blood from non-fasting participants at each sampling round. Serum samples were analyzed at the CDC for 11 PBDE congeners (BDE-17, BDE-28, BDE-47, BDE-66, BDE-85, BDE-99, BDE-100, BDE-153, BDE-154, BDE-183, BDE-209) using established methods (Sjödin et al. 2004). The coefficient of variation (CV) of included quality control (QC) samples was < 10% (data not shown). The limits of detection (LODs) of the PBDE congeners ranged from 0.2 to 0.8 ng/g lipid. Serum samples were analyzed for total triglycerides (triglycerides, GPO-PAP) and total cholesterol (cholesterol, CHOD-PAP) using colorimetric test kits from Roche Diagnostics at the CDC. Final determinations were made on a Hitachi Modular P Chemistry Analyzer. The concentration of total lipids was calculated by summation of the individual lipid components (Phillips et al. 1989).

Serum TFTs were measured at the Boston University School of Medicine. Enzyme-linked immunosorbent assays (ELISAs) were used to measure serum TSH (normal range: 0.4–4.2 mIU/L), fT_4_ (normal range: 0.8–2.0 ng/dL), TT_3_ (normal range 0.52–1.85 ng/mL), and TT_4_ (normal range 4.4–10.8 μg/dL in men and 4.8–11.6 μg/dL in women) (Immuno-Biological Laboratories Inc.). Thyroid peroxidase (TPO) antibodies were measured using an immunometric enzyme immunoassay (Orgentec Diagnostika) with the following reference ranges: normal < 50 IU/mL; borderline 50–75 IU/mL, and elevated > 75 IU/mL.

*Urine samples.* We collected 90 mL of urine from the participants at each sampling round. Urinary specific gravity was measured using a refractometer. We measured levels of urinary iodide, urinary perchlorate, and urinary thiocyanate using ion chromatography–mass spectrometry (IC-MS) with established methods (Valentin-Blasini et al. 2005) at the Boston University School of Medicine. The LODs and CVs were 0.05 μg/L (CV = 2.2% to 5.9%), 10 μg/L (CV < 5%), and 0.05 mcg/L (CV < 5%) for perchlorate, thiocyanate, and iodide, respectively.

*Statistical analysis.* For PBDE measurements below the LOD, we substituted LOD/2. ΣPBDE was defined as the sum of the five most prevalent pentaBDE congeners: BDE-28, BDE-47, BDE-99, BDE-100, and BDE-153. We assessed the normality of PBDEs, TFTs, and covariates using histograms and Shapiro–Wilk tests. TSH, fT_4_, and TT_3_ were log-normally distributed and, therefore, were natural log–transformed for regression analysis. TT_4_ was normally distributed and was modeled without transformation (see Supplemental Material, Figure S1). We used Spearman’s correlation coefficient to assess associations between PBDE congeners. All statistical analyses were performed using SAS statistical software (v9.3; SAS Institute Inc.), and statistical significance is reported at the 0.05 level.

We used a general linear model with a random intercept to assess the association between PBDEs and TFTs, which in its simplest form is

*Y_ij_ =* β*_o_ +* β*_1_*PBDE*_ij_ + b_i_ +* ε*_ij_*, [1]

where *Y_ij_* represents the thyroid hormone level of the *i*th participant at the *j*th sampling round, β*_o_* is the fixed-effect intercept, β*_1_* is an estimate of the mean difference in the TFT (or ln-TFT) with a 1-unit change in PBDE concentration, *b_i_* is the random intercept of the *i*_th_ individual, and ε_ij_ is the random error. The following covariates were added to Equation 1: time (indicator variables for sampling round), total lipids (milligrams per deciliter), urinary iodine concentration (UIC) (micrograms per liter), age (years), sex (male/female), body mass index (BMI; kilograms per meter squared), TPO antibody (yes/no), oral contraceptives (yes/no), perchlorate (micrograms per liter), thiocyanate (micrograms per liter), and specific gravity (SG). We selected potential confounding variables based on the *a priori* expectation that they could be associated with the exposure and the outcome but not be on the causal pathway. We assessed confounding using a change of 10% or more in the PBDE beta coefficient as a guide. We ran regression models on data sets excluding women taking oral contraceptives (*n* = 7) and participants who tested positive for the TPO antibody (*n* = 3) to assess their potential to affect regression estimates. We assessed effect measure modification by stratification on the following variables: sex and UIC (< 100 μg/L vs. ≥ 100 μg/L). For continuous covariates, we assessed possible effect modification by including a cross product of PBDE level and covariate (total lipids, iodine, age, BMI).

We conducted regression analyses between PBDEs and TFTs with (51 participants, 137 serum samples) and without (50 participants, 134 serum samples) a participant who had experienced very high PBDE exposure and was identified as influential in this analysis with a Cook’s distance of 13.

We estimated intraclass correlation coefficients (ICCs) to assess the stability of TFTs and UIC using an intercept-only general linear model with a random intercept. The ICCs were estimated by dividing the between-subject variance by the total variance.

## Results

The final study population for the thyroid analysis consisted of 26 males and 25 females. Participation rate by sampling round was 96% in round 1 (winter 2010), 96% in round 2 (summer 2011), and 81% in round 3 (winter 2011). The median age was 37 years old; 98% of the participants had a college degree; and 63% of the participants had BMI < 25 kg/m^2^ (Table 1). Forty-five participants identified as white non-Hispanic, 3 as Hispanic/Latino, and 3 as Asian. Table 2 presents the round-specific geometric means (GMs), geometric standard deviations (GSDs), detection frequencies, and ranges for PBDE congeners that were detected in > 50% of serum samples: BDE-28, BDE-47, BDE-99, BDE-100, and BDE-153. Detection rates for other PBDE congeners were low, and these results were not analyzed further (Makey et al. 2014). GM concentrations and ranges of ΣPBDE in sampling rounds 1, 2, and 3 were 22 (2.3–290) ng/g lipid, 23 (2.7–290) ng/g lipid, and 19 (2.4–210) ng/g lipid, respectively. BDE-47 was the predominant serum congener. BDE-28, BDE-47, BDE-99, and BDE-100 were highly and significantly correlated (*r* ≥ 0.85 for concentrations measured at the first visit), whereas BDE-153 was moderately correlated with the lower brominated PBDE congeners (*r* = 0.29–0.47) (see Supplemental Material, Table S1).

**Table 1:** Baseline characteristics, collected in 2010, from the FlaRE cohort (51 participants).

Characteristic	*n* (%)
Age (years)
20–39	29 (57)
40–59	18 (35)
≥ 60	4 (8)
Sex
Female	25 (49)
Male	26 (51)
Race/ethnicity
White	45 (88)
Other	6 (12)
Education
College graduate	50 (98)
< College graduate	1 (2)
BMI (kg/m^2^)
< 25	33 (63)
25–29.9	16 (33)
≥ 30	2 (4)
Abbreviations: BMI, body mass index; FlaRE, Flame Retardant Exposure Study.	

**Table 2:** Descriptive statistics of analytes by sampling round (51 participants, 137 serum samples).

Analytes	Round 1 (*n *= 47)				Round 2 (*n *= 49)				Round 3 (*n *= 41)			
	GM	(GSD)	%	Range	GM	(GSD)	%	Range	GM	(GSD)	%	Range
Serum PBDEs (ng/g lipid)
∑PBDE^*a*^	22	(2.7)	100	2.3–290	23	(2.4)	100	2.7–290	19	(2.1)	100	2.4–210
BDE-28^*a*^	0.57	(2.3)	66	0.20–4.3	0.60	(2.2)	71	0.20–5.1	0.50	(2.2)	66	0.20–3.6
BDE-47^*a*^	9.5	(2.9)	100	0.60–150	9.9	(2.7)	100	1.3–150	7.6	(2.6)	100	0.90–99
BDE-99^*a*^	1.8	(3.0)	94	0.20–44	1.9	(3.0)	90	0.20–34	1.7	(2.5)	95	0.20–20
BDE-100^*a*^	1.8	(3.5)	87	0.20–42	1.9	(3.3)	90	0.20–44	1.4	(3.0)	90	0.20–35
BDE-153^*a*^	6.4	(3.2)	100	0.60–97	6.7	(3.1)	100	0.70–95	5.3	(3.3)	100	0.10–55
Serum PBDEs (pg/g serum)
∑PBDE^*a*^	130	(2.7)	100	14–1,900	140	(2.7)	100	15–2,000	120	(2.4)	100	15–1,500
BDE-28^*a*^	3.5	(2.4)	66	1.3–29	3.7	(2.4)	71	1.3–30	3.3	(2.3)	66	1.3–25
BDE-47^*a*^	57	(3.0)	100	3.9–990	59	(2.8)	100	7.3–1,000	48	(2.7)	100	5.5–710
BDE-99^*a*^	11	(3.0)	94	1.3–290	11	(3.2)	90	1.3–230	10	(2.6)	95	1.3–140
BDE-100^*a*^	11	(3.1)	87	1.3–280	11	(3.4)	90	1.3–300	8.0	(3.1)	90	1.3–250
BDE-153^*a*^	38	(3.6)	100	3.9–700	39	(3.1)	100	4.5–580	36	(2.7)	100	5.3–400
Thio (μg/L)^*a*^	450	(470)	100	72–2,300	570	(360)	100	61–1,500	480	(450)	100	16–1,800
Perchl (μg/L)^*a*^	3.8	(4.3)	100	0.40–19	2.9	(18)	100	0.40–130	5.2	(11)	100	0.40–73
TT_4_ (μg/dL)^*b*^	7.3	(1.3)	100	3.2–9.6	7.3	(1.4)	100	3.9–10	7.1	(1.3)	100	4.7–9.2
fT_4_ (ng/dL)^*a*^	1.2	(1.2)	100	0.90–2.1	1.2	(1.2)	100	1.0–2.2	1.2	(1.2)	100	1.0–1.6
TSH (μIU/mL)^*a*^	0.71	(1.8)	100	0.20–2.7	0.75	(1.8)	100	0.25–2.3	0.89	(1.9)	100	0.18–4.8
TT_3_ (ng/mL)^*a*^	1.1	(1.2)	100	0.78–1.5	1.2	(1.2)	100	0.80–1.4	1.2	(1.2)	100	0.82–1.5
UIC (μg/L)^*a*^	130	(170)	100	27–890	140	(100)	100	11–550	150	(116)	100	32–660
Urinary SG^*b*^	1.02	(0.007)	100	1.00–1.03	1.02^*d*^	(0.009)	100	1.00–1.04	1.02	(0.007)	100	1.00–1.03
TPO Ab (IU/mL)^*c*^	3	NA	6	< 5.0–810	3.0	NA	6	< 5.0–680	3.0	NA	7	< 5.0–640
Chol (mg/dL)^*b*^	190	(34)	100	120–290	190	(46)	100	110–360	190	(44)	100	92–330
Trig (mg/dL)^*b*^	130	(70)	100	50–340	130	(71)	100	42–340	140	(66)	100	45–290
Lipids (mg/dL)^*b*^^,^^*e*^	620	(110)	100	450–860	620	(150)	100	370–1,100	630	(140)	100	330–1,000
Abbreviations: %, percent detected; BDE, bromodiphenyl ether; Chol, cholesterol; fT_4_, free thyroxine; GM, geometric mean; GSD, geometric standard deviation; Lipids, total lipids; NA, not applicable; SG, specific gravity; PBDE; pentabromodiphenyl ether; Perchl, perchlorate; Thio, thiocyanate; TPO Ab, thyroid peroxidase antibody; Trig, triglycerides; TSH, thyroid-stimulating hormone; TT_3_, total triiodothyronine; TT_4_, total thyroxine; UIC, urinary iodine concentration. ^***a***^Geometric mean and geometric standard deviation. ^***b***^Mean and standard deviation. ^***c***^Number of participants who tested positive for TPO Ab in each round. ^***d***^One specific gravity measurement was missing in round 2. ^***e***^Total lipids = [cholesterol (mg/dL) × 2.27] + triglycerides (mg/dL) + 62.3.												

Table 2 presents the measures of central tendency and the ranges of the TFT values for TT_4_, TSH, fT_4_, TT_3_ and TPO. TFTs were predominantly within normal ranges (data not shown). Three female participants (ages 37, 54, and 57 years old) had elevated TPO antibodies, which are markers for thyroid autoimmunity, but their TFT values were within normal ranges (data not shown). A sensitivity analysis that excluded these three participants did not alter the results (data not shown). The median UICs by sampling round were all > 100 μg/L, indicative of iodine sufficiency [World Health Organization (WHO) 2013].

Significant, inverse associations between PBDEs and serum TT_4_ were estimated in this cohort of North American office workers (Table 3). Adjusting for sampling round, blood lipid level, age, sex, BMI, UIC, urinary specific gravity, urinary perchlorate, and urinary thiocyanate (model C), we found that for every 1-unit increase in BDE-47, there was a 2.6-μg/dL [95% confidence interval (CI): –4.7, –0.35] decrease in TT_4_ (*p* = 0.02). Additionally, we found that for every unit increase in BDE-100, there was a 7.8-μg/dL (95% CI: –14, –1.6) decrease in TT_4_ (*p* = 0.01). Estimates from the crude (model A) and lipid-adjusted (model B) models also indicated inverse associations between PBDEs and TT_4_ (Table 3). Associations between TT_4_ and BDE-28, BDE-99, and BDE-153 were also inverse, but only some of the associations were statistically significant (Table 3). Unadjusted cross-sectional associations between PBDEs and TT_4_ at each sampling round were consistently negative (data not shown). In the Supplemental Material, Figure S2A,B presents the unadjusted, cross-sectional negative association between BDE-47 and TT_4_ in round 1.

**Table 3:** Results from general linear regression models evaluating the association between PBDEs (ng/g serum) and thyroid function tests (51 participants, 137 serum samples).

TFT/Model	BDE-28 β (95% CI)	*p-*Value	BDE-47 β (95% CI)	*p-*Value	BDE-99 β (95% CI)	*p-*Value	BDE-100 β (95% CI)	*p-*Value	BDE-153 β (95% CI)	*p-*Value
TT_4_ (μg/dL)
Model A	–51 (–110, 6.2)	0.08	–2.7 (–4.8, –0.61)	0.01	–7.3 (–15, 0.042)	0.05	–8.4 (–14, –2.5)	0.01	–2.8 (–5.5, 0.04)	0.05
Model B	–33 (–95, 29)	0.29	–2.3 (–4.5, –0.18)	0.03	–6.4 (–14, 0.93)	0.09	–7.5 (–14, –1.4)	0.02	–2.4 (–5.2, 0.43)	0.10
Model C	–34 (–97, 28)	0.28	–2.6 (–4.7, –0.35)	0.02	–7.6 (–15, –0.06)	0.05	–7.8 (–14, –1.6)	0.01	–2.3 (–5.2, 0.61)	0.12
TSH ln(μIU/mL)^*a*^
Model A	0.29 (–28, 28)	0.99	0.32 (–0.71, 1.4)	0.54	1.6 (–2.1, 5.3)	0.39	1.3 (–1.6, 4.2)	0.37	0.97 (–0.34, 2.3)	0.15
Model B	–11 (–41, 18)	0.45	0.10 (–0.95, 1.2)	0.85	1.2 (–2.6, 4.9)	0.54	0.84 (–2.1, 3.8)	0.58	0.83 (–0.49, 2.2)	0.21
Model C	–11 (–42, 20)	0.49	0.17 (–0.92, 1.3)	0.75	1.5 (–2.5, 5.4)	0.46	0.92 (–2.2, 4.0)	0.55	0.81 (–0.58, 2.2)	0.25
fT_4_ ln(ng/dL)^*a*^
Model A	–0.40 (–7.5, 6.7)	0.92	–0.13 (–0.39, 0.1)	0.35	–0.72 (–1.7, 0.23)	0.14	0.27 (–0.47, 1.0)	0.47	0.40 (–0.09, 0.72)	0.01
Model B	1.1 (–6.6, 8.9)	0.77	–0.10 (–0.4, 0.17)	0.46	–0.67 (–1.6, 0.30)	0.17	0.35 (–0.41, 1.1)	0.37	0.42 (0.10, 0.75)	0.01
Model C	0.42 (–7.3, 8.1)	0.91	–0.13 (–0.40, 0.1)	0.34	–0.83 (–1.8, 0.14)	0.09	0.21 (–0.54, 0.95)	0.58	0.35 (0.03, 0.67)	0.04
TT_3_ ln(ng/mL)^*a*^
Model A	0.01 (–0.3, 0.3)	0.99	–0.01 (–0.3, 0.3)	0.94	0.12 (–0.91, 1.1)	0.82	–0.28 (–1.0, 0.47)	0.46	–0.28 (–0.6, 0.05)	0.10
Model B	2.2 (–5.9, 10)	0.60	0.03 (–0.25, 0.3)	0.82	0.23 (–0.81, 1.3)	0.67	–0.20 (–0.98, 0.6)	0.61	–0.27 (–0.6, 0.08)	0.13
Model C	2.2 (–5.9, 10)	0.62	0.04 (–0.24, 0.3)	0.77	0.21 (–0.84, 1.3)	0.69	–0.13 (–0.9, 0.64)	0.73	–0.19 (–0.54, 0.2)	0.28
Abbreviations: β, beta-estimate; BDE, bromodiphenyl ether; CI, confidence interval; fT_4_, free thyroxine; *p*, *p*-value; TFT, thyroid function test; TT_3_, total triiodothyronine; TT_4_, total thyroxine; TSH, thyroid-stimulating hormone; SG, specific gravity. Model A: Exposure only, no covariates. Model B: Adjusted for serum lipids only. Model C: Adjusted for serum lipids, age, sex, BMI, urine iodide, urine perchlorate, urine thiocyanate, and urine specific gravity. All covariates other than sex were modeled as untransformed continuous variables. ^***a***^Dependent variables are natural log–transformed.										

We identified a possible influential point in the relationship between PBDEs and TT_4_. The participant was a 64-year-old, white, non-Hispanic male with high exposure levels of BDE-47 (approximately 13–15 times the FlaRE BDE-47 population sampling round geometric means). When this point was omitted in regression analysis, the inverse relationships between PBDEs and TT_4_ were larger (data not shown).

Table 3 also presents the associations between PBDEs and the following TFT outcomes: TSH, fT_4_, and TT_3_. These three outcomes were non-normally distributed and were natural log (ln)–transformed for regression analysis (see Supplemental Material, Figure S1). The associations of PBDEs with TSH were positive but small and not statistically significant. We did not find any important associations between PBDEs and fT_4_ or TT_3_. We estimated an inverse association between BDE-153 and ln(TT_3_) (95% CI: –0.54, 0.20). We report a positive, significant association between BDE-153 and ln(fT_4_) (95% CI: 0.03, 0.67). However, after removal of the influential participant (three serum samples), the positive association between BDE-153 and fT_4_ was attenuated (β = 0.03; 95% CI: –0.08, 0.13). There were no consistent associations between the lower brominated PBDEs and fT_4_.

For the primary models described above (models A, B, and C) we included PBDEs on a wet weight basis (nanograms per gram serum), treating lipids as a covariate in our regression models, which allowed us to assess the independent effects of PBDEs and serum lipids. In almost all cases, adjusting for serum lipids affected the beta coefficients (> 10% change). In contrast, adjusting for demographic and other covariates (e.g., age, sex, BMI, urinary iodine, urinary specific gravity, urinary perchlorate, urinary thiocyanate) did not affect our results as strongly. We ran regression models standardizing PBDEs to lipids, and the inverse relationships between PBDEs and TT_4_ were persistent (see Supplemental Material, Table S2).

Our results were not confounded (e.g., no confounding if < 10% change in the point estimate) by urinary iodide (data not shown). When we stratified our cohort by sex and ran regression models on data sets that excluded women taking oral contraceptives (seven women), or participants who were TPO-antibody positive (three women), we observed an inverse association between PBDEs and TT_4_ (data not shown). We did not find statistically significant interaction terms for PBDE level with age, sex, BMI, or total lipids (data not shown). Inverse relationships between PBDEs and TT_4_ were persistent and similar in groups dichotomized by iodide status at the first round of sampling: UIC < 100 μg/L versus UIC ≥ 100 μg/L (data not shown).

The ICCs for the TFTs were TSH = 0.72 (95% CI: 0.56, 0.78); TT_4_ = 0.80 (95% CI: 0.68, 0.85); fT_4_ = 0.69 (95% CI: 0.53, 0.77); TT_3_ = 0.56 (95% CI: 0.39, 0.67) (see Supplemental Material, Table S3). The ICCs estimated in our cohort were similar to those reported in an earlier study of monthly variation in TFTs among 15 healthy, Caucasian men (Andersen et al. 2002).

## Discussion

Our study population of 51 adults living in the Greater Boston metropolitan area was iodide sufficient and did not have overt thyroid dysfunction. Using repeated serum measures in our prospective cohort, we found an inverse association between pentaBDE congeners and TT_4_. The inverse association between PBDEs and TT_4_ was persistent regardless of the method of lipid adjustment and in regression models adjusted for potential confounders. Our results are consistent with animal studies showing that exposure to pentaBDEs often caused a reduction in TT_4_ (Dishaw et al. 2014). TSH, fT_4_, and TT_3_ concentrations were not clearly associated with PBDE exposures in our study population of healthy adults, which suggests that associations with TT_4_ are not a consequence of effects on the hypothalamic–pituitary–thyroid (HPT) axis.

PBDEs and their hydroxylated metabolites (OH-PBDEs) are structurally similar to the thyroid hormones T_4_ and T_3_ (Meerts et al. 2000). Therefore, PBDEs have been extensively investigated as possible TH disruptors in animal studies. Many *in vivo* toxicological studies in rodents have reported a reduction in TT_4_ following exposures to commercial PBDE (e.g., DE-71) or to individual congeners (Blanco et al. 2013; Ernest et al. 2012; Fowles et al. 1994; He et al. 2011; Kodavanti et al. 2010; Stoker et al. 2004; van der Ven et al. 2008; Zhou et al. 2001, 2002) except for one study that showed an increase in TT_4_ (Blake et al. 2011). Decreases in TT_4_ have been observed in rodents under multiple exposure protocols (acute, sub-acute, chronic) and at various developmental time points (prenatal, perinatal, postnatal, adolescent, adult). Some animal exposure experiments have shown decreases in TT_3_ (Blanco et al. 2013; Stoker et al. 2004; Zhou et al. 2001); however, T_3_ (total or free) was not always measured in *in vivo* rodent studies (Ernest et al. 2012; Fowles et al. 1994), or the decrease in T_3_ was not significant (He et al. 2011; van der Ven et al. 2008; Zhou et al. 2002). The reported effects of PBDEs on TSH have been less consistent. Two *in vivo* rodent studies reported an increase in TSH in response to PBDE exposure (Stoker et al. 2004; Ellis-Hutchings et al. 2006), which would be indicative of PBDEs having an effect on the HPT axis. In contrast, four *in vivo* rodent studies have reported a decrease in TT_4_ without an increase in TSH (Ernest et al. 2012; He et al. 2011; Zhou et al. 2001, 2002).

A decrease of peripheral THs (TT_4_ or TT_3_) in animals in response to PBDE exposures has prompted investigations of the possible underlying mechanisms for this phenomenon. *In vitro* experiments have shown that some OH-PBDEs interact with the serum thyroid hormone binding protein transthyretin (TTR), possibly displacing T_4_ from TTR (Marchesini et al. 2008; Meerts et al. 2000). Additionally, BDE-47 may alter T_4_ transport by affecting hepatic *TTR* mRNA expression, decreasing the amount of binding protein available (Richardson et al. 2008). *In vivo* experiments have shown that exposure to commercial pentaBDE mixtures (e.g., DE-71) leads to an induction of uridine diphosphate glucuronosyltransferase, which may increase T_4_-glucuronidation and deplete circulating T_4_, leading to decreased TT_4_ levels (Stoker et al. 2004; Zhou et al. 2002). The mechanism(s) underlying TH disruption by PBDEs in animals has not been completely elucidated and is likely multifactorial. Nevertheless, toxicological studies to date have consistently shown that exposure to pentaBDE mixtures or individual congeners leads to a decrease in serum TT_4_ concentration.

T_4_ exists in two forms: approximately 99.97% of circulating T_4_ is bound to plasma proteins [thyroxine-binding globulin (TBG), TTR, and albumin]; the remaining T_4_ (fT_4_) is unbound and available for deiodination in the outer phenolic ring to generate the bioactive hormone T_3_ (Benvenga 2013; Bianco and Kim 2013). In humans, measurements of fT_4_ have mostly replaced measurements of TT_4_ (which is predominately bound hormone) as a measure of thyroid status in clinical practice (Garber et al. 2012). Abnormally high or low TT_4_ is affected by factors involving TH serum transport proteins and is not necessarily indicative of thyroid dysfunction (Garber et al. 2012). Typically, TT_4_ is decreased if substances are present that can displace TH from protein-binding sites (Stockigt and Lim 2009), or if there is a decrease in the TH transport proteins, mainly TBG (De Groot et al. 2012). We report that serum PBDE concentrations were inversely associated with serum TT_4_ in our study population of healthy adults. Two possible hypotheses are that PBDEs displace THs from their transport proteins or that PBDEs decrease the amount of plasma-binding proteins, resulting in a decrease in TT_4_. However, it should be emphasized that comparing T_4_ binding to plasma proteins in rodents with that in humans may present a challenge because the major binding protein in rodents is TTR, and the major binding protein in humans is TBG (Choksi et al. 2003).

Epidemiological studies have reported associations between PBDEs and TFTs, but the results have been inconsistent. Although our results showing inverse associations between PBDEs and TT_4_ are consistent with those typically observed in animal experiments and in some human studies (Abdelouahab et al. 2013; Herbstman et al. 2008; Lin et al. 2011), they differ from other studies that have reported positive associations with TT_4_. Turyk et al. reported that serum PBDEs were positively associated with serum TT_4_ and urinary T_4_ in a cohort of 405 adult men who consumed sport fish (Turyk et al. 2008). However, these authors also reported that PBDEs were related to the percentage of TBG bound to T_4_ and to an increase in the percentage of TT_4_ bound to albumin, indicating that PBDEs may affect serum binding patterns (Turyk et al. 2008). Two studies of pregnant women reported that PBDE exposure was inversely associated with TSH; one was a study of 207 women with measurements obtained at approximately 27 weeks of gestation (Chevrier et al. 2010), and the other was a study of 25 women with samples collected during the second trimester (Zota et al. 2011). A study of 140 women with samples collected after 34 weeks of gestation reported a positive association between PBDE exposure and the thyroid hormones TT_4_ and fT_4_ (Stapleton et al. 2011). It is difficult to compare the results for pregnant women to those obtained in our study because TT_4_ is increased by up to 50% in the first trimester of pregnancy because of estrogen-induced elevations of serum TBG (Stagnaro-Green et al. 2011), which can increase TT_4_ levels.

At the present time, there is debate regarding how (or whether) to adjust for serum lipids—lipids as a covariate, lipid-standardization—when studying the health effects of lipophilic chemicals (Schisterman et al. 2005). As expected, serum PBDE concentrations were positively associated with serum lipid concentrations in our population (Makey et al. 2014). Additionally, thyroid hormones maintain lipid homeostasis by affecting gene expression in adipose tissue and the liver, which in turn affects lipolysis and clearance (Pearce 2012), thus making serum lipids dependent on the thyroid hormones. The causal structure among PBDEs, serum lipids, and THs is unclear at present. Therefore, as recommended elsewhere, we compared multiple methods for lipid adjustment (Chevrier 2013; Schisterman et al. 2005). We found that the results obtained from our crude and lipid-adjusted models were generally consistent in the direction of the association, but the magnitudes of the β-coefficients were altered by > 10%. The direction of the associations between PBDEs and TT_4_ remained the same between the lipid-adjusted and the lipid-standardized models. Comparison between these models is difficult to make because the denominators for the β-coefficients are different.

Iodide, an essential component of THs, has been reported to be an effect measure modifier in epidemiologic studies such that subgroups with insufficient iodine intake appear to be more vulnerable to xenobiotic effects on TH levels than iodine-sufficient subgroups (Blount et al. 2006). Iodide did not appear to modify associations between PBDEs and TT_4_ in our iodine-sufficient study population.

A major strength of our study was the use of three serum samples from a longitudinal cohort free of overt thyroid dysfunction. We collected demographic and medical information to assess confounding and effect measure modification. Our study, however, also had some limitations. We did not measure OH-PBDE metabolites, which have been shown to have binding affinities for TBG and TTR (Marchesini et al. 2008). We did not have three serum samples from all participants. Selected samples were excluded based on *a priori* criteria (medication use, pregnancy, inadequate serum volume for chemical analysis), and some samples were missing because participants completed only one (*n* = 2) or two (*n* = 9) of the three (*n* = 41) study visits, leaving a total of 137 serum samples from 51 participants. We believe that the likelihood that an individual sample was missing would have been unrelated to the actual TFT value of the missing sample, which would, on average, result in data that are missing completely at random (MCAR). If this assumption is correct for our population, missing data should not have biased associations because general linear models are robust to missing data that are MCAR (Little and Rubin 2002). Our study sample size was relatively small; we used a convenience sample of office workers in the Boston metropolitan area who were 85% white and highly educated, and we cannot be certain that our results can be generalized to the general U.S. adult population. Furthermore, our small sample size limited our ability to evaluate effect measure modification.

## Conclusion

The results from our repeated measures cohort study suggest that environmental exposure to PBDEs is associated with reduced TT_4_ levels. The lack of clear associations with other thyroid function parameters suggests that the negative association with TT_4_ might be a consequence of decreased serum binding of T_4_. This finding is consistent with the toxicological literature and with some human studies. Our conclusions were robust to potential confounders and to various methods of data analysis. Prospective studies are needed to further understand how PBDEs and their metabolites may affect TH homeostasis in healthy adults.

## Supplemental Material

(565 K B) PDF
