# Supplementary material for: Polybrominated Diphenyl Ether Exposure and Thyroid Function Tests in North American Adults
Source: Environ Health Perspect. 2015 Sep 15;124(4):420–5. doi: 10.1289/ehp.1509755 (PMC4829976; doi:10.1289/ehp.1509755)
Supplement: (565 K B) PDF [file ehp.1509755.s001.acco.pdf]

Environ Health Perspect
DOI: 10.1289/ehp.1509755

Note to Readers: *EHP* strives to ensure that all journal content is accessible to all readers. However, some figures and Supplemental Material published in *EHP* articles may not conform to 508 standards due to the complexity of the information being presented. If you need assistance accessing journal content, please contact ehp508@niehs.nih.gov. Our staff will work with you to assess and meet your accessibility needs within 3 working days.

Supplemental Material

Polybrominated Diphenyl Ether Exposure and Thyroid Function Tests in North American Adults

Colleen M. Makey, Michael D. McClean, Lewis E. Braverman, Elizabeth N. Pearce, Xue-Mei He, Andreas Sjödin, Janice M. Weinberg, and Thomas F. Webster

Table of Contents

Table S1. Spearman correlation coefficients between PBDE congeners at initial sampling round (47 serum samples).

Table S2. Results from general linear regression models evaluating the association between lipid-standardized PBDEs and thyroid function tests. (51 adults, 137 serum samples).

Table S3. Estimated ICCs and variance components for repeated serum markers of thyroid function tests.

Figure S1. Distribution of thyroid function tests: untransformed and natural log transformed: (A) Total T_4, (B) TSH, (C) Free T_4, (D) Total T_3.

Figure S2A-B. Unadjusted cross-sectional associations between BDE-47 and Total T_4 in Round 1: A) without outlier (N=46) B) with outlier (N=47)*.

Table S1. Spearman correlation coefficients between PBDE congeners at initial sampling round (47 serum samples).

	BDE-28	BDE-47	BDE-99	BDE-100	BDE-153
BDE-28	1.0	*r*=0.92<br>*p*<0.0001	*r*=0.88<br>*p*<0.0001	*r*=0.85<br>*p*<0.0001	*r*=0.29<br>*p*=0.045
BDE-47		1.0	*r*=0.97<br>*p*<0.0001	*r*=0.96<br>*p*<0.0001	*r*=0.42<br>*p*=0.0033
BDE-99			1.0	*r*=0.95<br>*p*<0.0001	*r*=0.40<br>*p*=0.0054
BDE-100				1.0	*r*=0.47<br>*p*=0.0008
BDE-153					1.0

Abbreviations: *p* (p-value), *r* (spearman correlation coefficient)

Table S2. Results from general linear regression models evaluating the association between lipid-standardized PBDEs and thyroid function tests. (51 adults, 137 serum samples).

TFTs	BDE-28 β (95% CI)	p	BDE-47 β (95% CI)	p	BDE-99 β (95% CI)	p	BDE-100 β (95% CI)	p	BDE-153 β (95% CI)	p
**TT_4 (µg/dL)**										
	-15 (-54, 23)	0.43	-13 (-27, 0.57)	0.06	-34 (-81, 13)	0.16	-48 (-90, -6.7)	0.02	-12 (-31, 6.8)	0.21
**TSH ln(µIU/mL)[a]**										
	47 (230, 130)	0.62	0.81 (-6.0, 7.6)	0.81	6.1 (-18, 30)	0.61	7.5 (-12, 14)	0.46	5.6 (-3.1, 14)	0.20
**fT_4 ln(ng/dL)[a]**										
	2.9 (-46, 51)	0.91	-0.57 (-2.3, 1.2)	0.53	-3.7 (-9.8, 2.5)	0.24	2.5 (-2.6, 7.5)	0.33	3.0 (0.87, 5.0)	0.01
**TT_3 ln(ng/mL)[a]**										
	6.1 (-44, 56)	0.81	0.15 (-1.7, 1.9)	0.87	1.4 (-5.3, 8.0)	0.68	-1.3 (-6.4, 3.8)	0.62	-1.5 (-3.7, 0.70)	0.18

Abbreviations: β (beta-estimate), CI (confidence interval), fT_4 (free thyroxine), p (p-value), TFTs (thyroid function tests), TT_3 (total triiodothyronine), TT_4 (total thyroxine), TSH (thyroid stimulating hormone)

a Dependent variable was natural log-transformed for regression analysis.

Table S3. Estimated ICCs and variance components for repeated serum markers of thyroid function tests.

Metrics	TSH (µIU/mL) Estimate (95% CI)	TT_4 Estimate (95% CI)	fT_4 Estimate (95% CI)	TT_3 Estimate (95% CI)	UIC Estimate (95% CI)
**ICC**	0.72 (0.56, 0.78)	0.80 (0.68, 0.85)	0.69 (0.53. 0.77)	0.56 (0.39, 0.67)	0.38 (0.19, 0.50)
σ^2_W	0.11 (0.09, 0.2)	0.36 (0.3, 0.5)	0.0075 (0.006, 0.01)	0.012 (0.009, 0.02)	0.38 (0.29, 0.53)
σ^2_B	0.28 (0.2, 04)	1.4 (0.8, 2.0)	0.017 (0.009, 0.03)	0.015 (0.007, 0.03)	0.23 (0.077, 0.38)

Abbreviations: ICC (intraclass correlation coefficient), TSH (thyroid stimulating hormone), TT_4 (total thyroxine), fT_4 (free thyroxine), TT_3 (total triiodothyronine), UIC (urinary iodide concentration), σ^2_W (within subject variability), σ^2_B (between subject variability)

Figure S1. Distribution of thyroid function tests: untransformed and natural log transformed: (A) Total T_4, (B) TSH, (C) Free T_4, (D) Total T_3.

A.

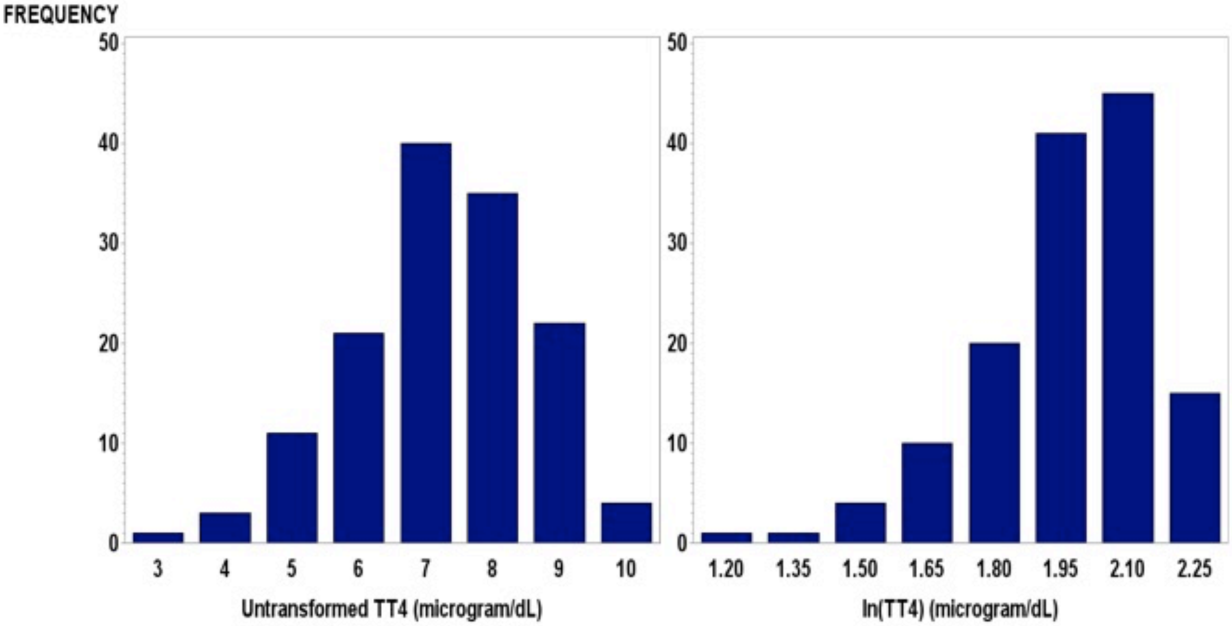

B.

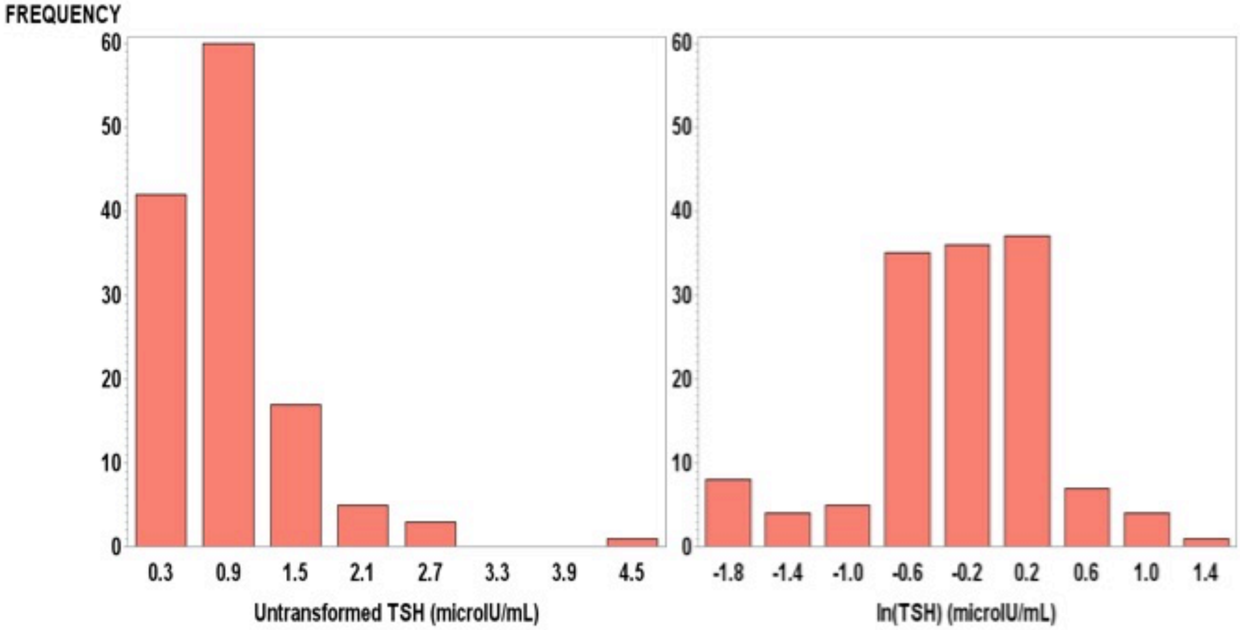

C.

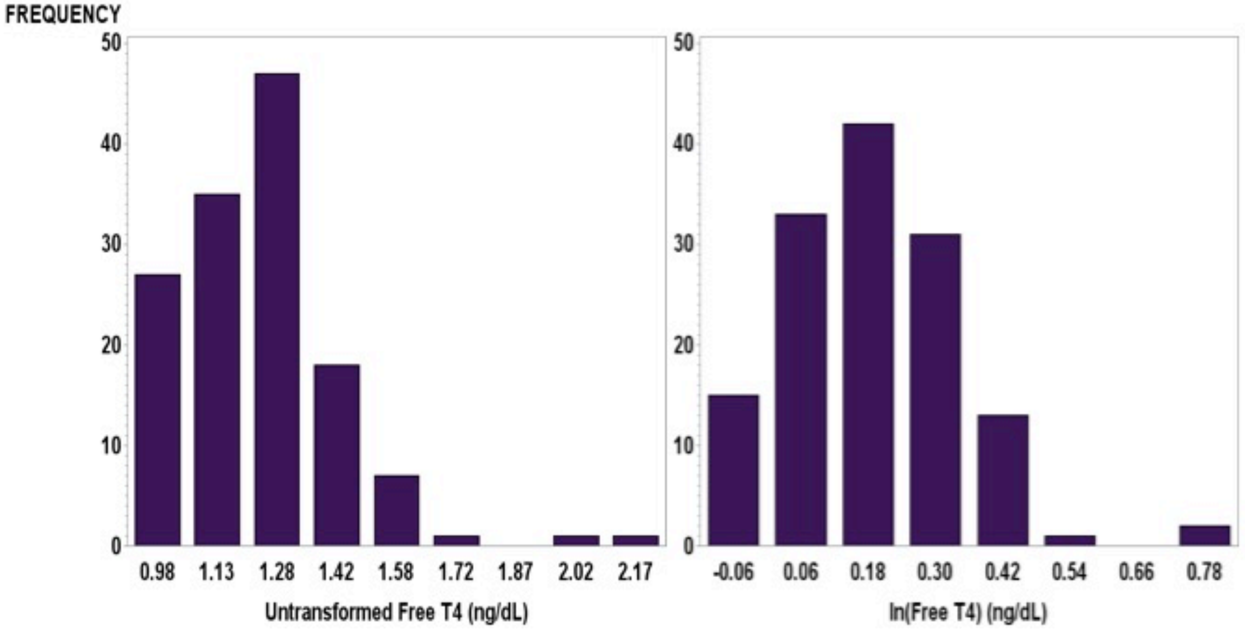
FREQUENCY
50
40
30
20
10
0
0.98
1.13
1.28
1.42
1.58
1.72
1.87
2.02
2.17
Untransformed Free T4 (ng/dL)
-0.06
0.06
0.18
0.30
0.42
0.54
0.66
0.78
ln(Free T4) (ng/dL)

D.

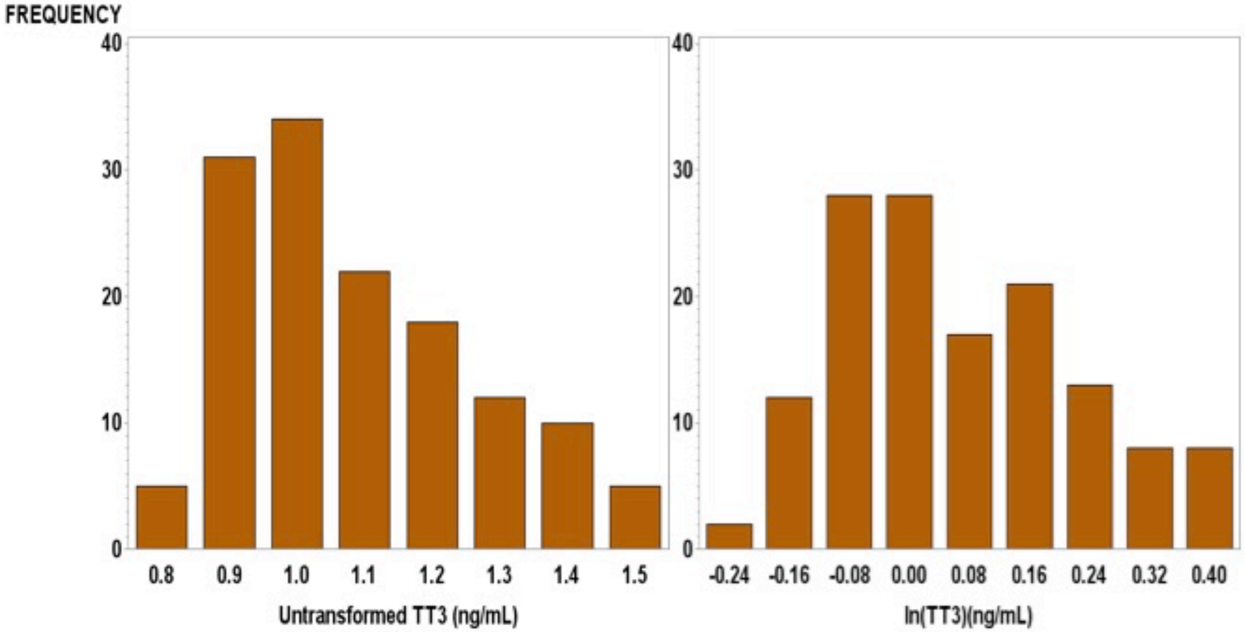
FREQUENCY
40
30
20
10
0
0.8
0.9
1.0
1.1
1.2
1.3
1.4
1.5
Untransformed TT3 (ng/mL)
-0.24
-0.16
-0.08
0.00
0.08
0.16
0.24
0.32
0.40
ln(TT3)(ng/mL)

Figure S2A-B. Unadjusted cross-sectional associations between BDE-47 and Total T_4 in Round 1: A) without outlier (N=46) B) with outlier (N=47)*.

A)

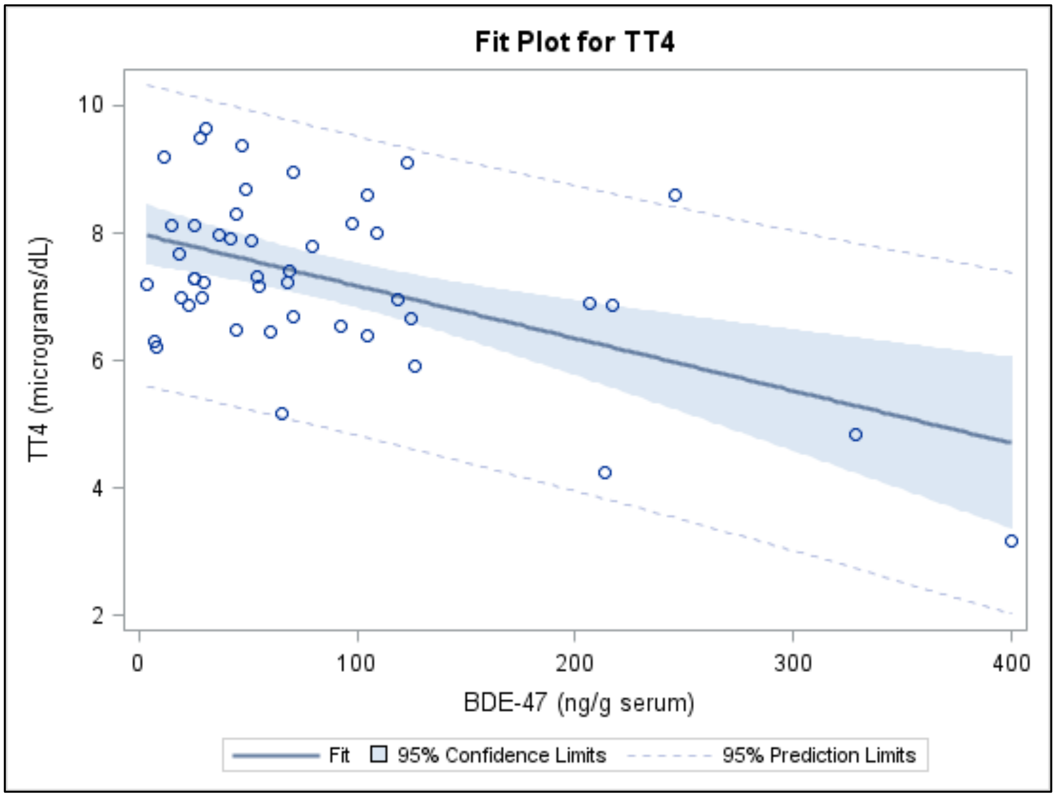

B.

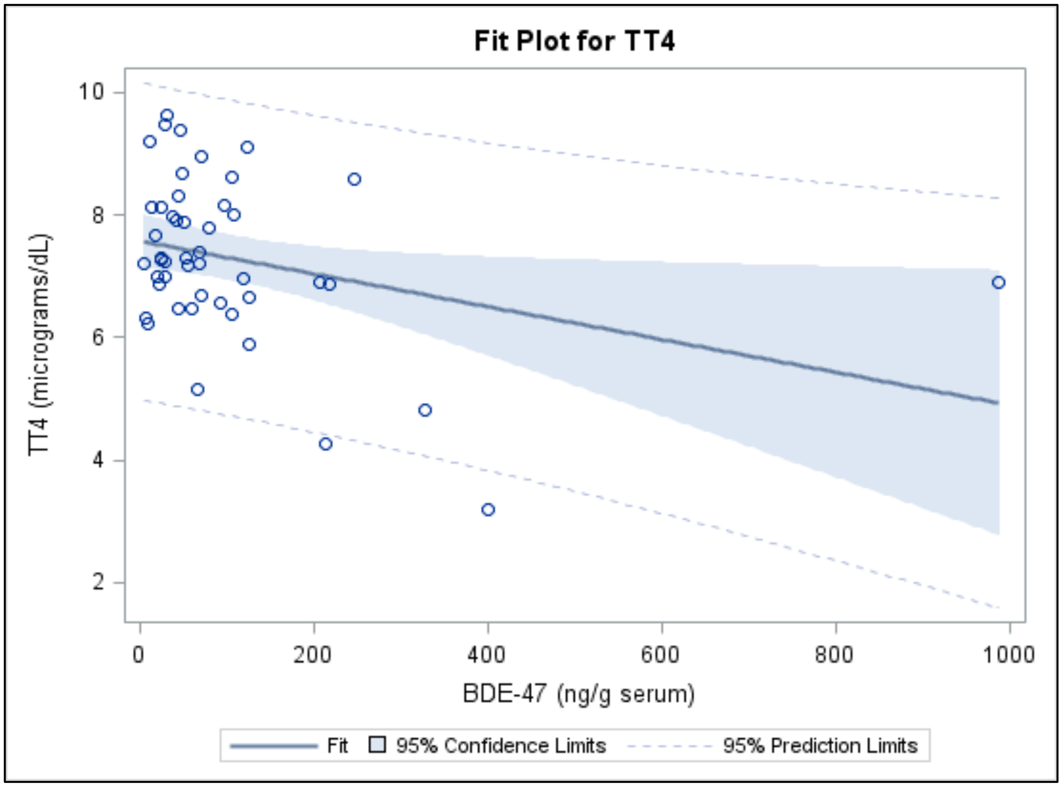

A) Relationship between BDE-47 and Total T_4 in Round 1 without outlying participant (N=46) (β=-8.2, 95% CI: -12.3, -4.1). B) Relationship between BDE-47 and Total T_4 in Round 1 with outlying participant (N=47) (β=-2.7, 95% CI: -5.1, -0.27). * Outlying participant was a 64 year old, non-Hispanic white male.
